# Polysialic acid is released by human umbilical vein endothelial cells (HUVEC) in vitro

**DOI:** 10.1186/s13578-018-0262-y

**Published:** 2018-12-11

**Authors:** Sebastian Strubl, Uwe Schubert, Andrea Kühnle, Alexander Rebl, Negah Ahmadvand, Silvia Fischer, Klaus T. Preissner, Sebastian P. Galuska

**Affiliations:** 1 Institute of Biochemistry, Faculty of Medicine, Justus-Liebig-University, Friedrichstrasse 24, 35392 Giessen, Germany; 2 Department II of Internal Medicine, Center for Molecular Medicine Cologne, University Cologne, Kerpener Str. 62, 50931 Cologne, Germany; 3 Institute of Reproductive Biology, Leibniz Institute for Farm Animal Biology (FBN), Wilhelm-Stahl-Allee 2, 18196 Dummerstorf, Germany; 4 Institute of Genome Biology, Leibniz Institute for Farm Animal Biology (FBN), Wilhelm-Stahl-Allee 2, 18196 Dummerstorf, Germany; 5 Excellence Cluster Cardio Pulmonary System (ECCPS), Aulweg 130, 35392 Giessen, Germany

**Keywords:** Human umbilical vein endothelial cells, Polysialic acid, Vascular endothelial growth factor

## Abstract

**Background:**

Sialic acids represent common terminal residues on numerous mammalian glycoconjugates, thereby influencing e.g. lumen formation in developing blood vessels. Interestingly, besides monosialylated also polysialylated glycoconjugates are produced by endothelial cells. Polysialic acid (polySia) is formed in several organs during embryonal and postnatal development influencing, for instance, cell migration processes. Furthermore, the function of cytokines like basic fibroblast growth factor (bFGF) is modulated by polySia.

**Results:**

In this study, we demonstrated that human umbilical vein endothelial cells (HUVEC) also secrete polysialylated glycoconjugates. Furthermore, an interaction between polySia and vascular endothelial growth factor (VEGF) was observed. VEGF modulates like bFGF the migration of HUVEC. Since both growth factors interact with polySia, we examined, if polySia modulates the migration of HUVEC. To this end scratch assays were performed showing that the migration of HUVEC is stimulated, when polySia was degraded.

**Conclusions:**

Since polySia can interact with bFGF as well as VEGF and the degradation of polySia resulted in an increased cell migration capacity in the applied scratch assay, we propose that polySia may trap these growth factors influencing their biological activity. Thus, polySia might also contribute to the fine regulation of physiological processes in endothelial cells.

## Introduction

Eukaryotic cells are generally covered by a glycocalyx, varying in thickness and composition and consisting of a broad range of glycoconjugates, including glycolipids, glycoproteins and proteoglycans [1]. Most of these glycoconjugates are sialylated at the distal end of the respective carbohydrate chains [2, 3]. Because of their terminal position as a negatively charged moiety on glycans, sialic acids play an important role during numerous physiological processes [2–5], such as the formation of new blood vessels [6]. While the majority of glycoconjugates contains monosialylated carbohydrate chains, some proteins are posttranslationally modified by polysialic acid (polySia) [7]. These sialic acid polymers are especially present throughout the development on cells of the brain [8, 9], but are also recognized in other organs like the female and male reproductive tract, where polySia-positive cells appear in a spatial–temporal manner during embryonal and postnatal development [10–16]. Due to their presence on adjacent cells, polyanionic polySia can modulate cell–cell interactions in a repulsive fashion during organogenesis [17–19]. This effect of polySia supports also metastasis of polySia positive tumors like neuroblastoma [20].

Moreover, interactions of polySia with basic proteins such as brain-derived neurotrophic factor (BDNF) or basic fibroblast growth factor (bFGF) [21] not only concentrate these proteins on the cell surface, but also modulate their cytokine function (enhancing BDNF; attenuating bFGF) [22, 23].

Recently, Naftolin and colleagues described a polysialylated form of the neural cell adhesion molecule (NCAM), expressed in rat and human endothelial cells [24–26]. Interestingly, they observed an estradiol-dependent production of polySia-NCAM and hypothesized whether the observed estrogen-induced cardio-protection was supported by polySia due to its repulsive properties in cell–cell interactions. In the present study, the biosynthesis of polySia and its influence on human umbilical vein endothelial cells (HUVEC) was investigated with particular emphasis on the modulation of their migration capacity.

## Materials and methods

### Materials

PolySia-specific monoclonal antibody (mAb) 735 and endo-neuraminidase (endoN) were kindly provided by Martina Mühlenhoff (Medizinische Hochschule, Hannover, Germany) [27, 28]. For Western blot analysis, horseradish peroxidase-conjugated secondary antibodies were used (Dako, Hamburg, Germany). All reagents used were of analytical grade.

Umbilical cords were obtained from the Gynaecology Department, University Hospital Giessen (Germany) to isolate HUVEC. The local research ethics committee approved the use of HUVEC isolation (file reference 132/9).

### Human umbilical vein endothelial cells (HUVEC)

HUVEC were isolated according to the method by Jaffe et al. [29], and cells were cultured in endothelial cell growth medium (ECGM; PromoCell, Heidelberg, Germany) containing 10% FCS (PromoCell, Heidelberg, Germany). For all experiments, HUVEC in passages 2 to 4 were used. Since the cells may release polySia per se into the cell culture medium, HUVEC were washed twice and cultured overnight in endothelial cell basic medium (ECBM; PromoCell, Heidelberg, Germany) without FCS before the analysis of polySia in cell supernatants.

### Cell homogenization and purification of polysialylated proteins for Western blotting

Cells were washed three times with PBS and harvested with the help of a cell scraper. Cell homogenization was performed by using 50 mM Tris/HCl-buffer (pH 7.5) containing 1% Triton-X-100, 5% sodium deoxycholate, 50 mM EDTA, 150 mM NaCl, 2 mM PMSF, 1000 U/mL aprotinin and 1 mM leupeptin with the assistance of an ultrasonicator. Protein concentrations were determined with a BCA protein assay (ThermoFisher Scientific, Darmstadt, Germany).

To purify polySia-modified proteins, tosyl-modified magnetic dynabeads M-280 (Invitrogen, Darmstadt, Germany) were coated with anti-polySia antibody (10 µg mAb 735 for 50 µL Beads in 200 µL PBS-T), followed by incubation with homogenized samples. After several washing steps (3× washing buffer I: 20 mM Tris/HCl, pH 7.5 including 150 mM NaCl, 0.5% Triton-X-100) purified polysialylated proteins were eluted in 100 mM trimethylamine and the fractions were stored at − 20 °C.

For Western blotting, samples were subjected to SDS-PAGE on a 10% SDS-gel under reducing conditions (10 µL/lane), transferred to a PVDF membrane which was exposed to an anti-polySia antibody (mAb 735; 1 µg/mL), recognizing chain length with more than seven sialic residues, followed by incubation with a labeled secondary Ab and the chemiluminescence SuperSignal kit (ThermoFisher Scientific, Darmstadt, Germany). The PVDF membrane was developed with light sensitive Amersham Hyperfilm™ ECL (GE Healthcare Limited, Solingen, Germany) and developing solution. For negative control, samples were incubated with endoN (2 µg/mL, 1 h at 37 °C) prior to SDS-PAGE; this enzyme cleaves α2,8-linked polySia resulting in oligomers with chain length below seven sialic acid residues [27, 28].

### Immunofluorescence staining

HUVEC were transferred onto 4-well cell culture chamber slides (100,000 cells/well; Sarstedt, Nümbrecht, Germany) allowing to adhere for 24 h. Thereafter, cells were fixed and permeabilized with ice-cold 70% ethanol. In addition HUVC were fixed with 4% paraformaldehyde in PBS without an additional permeabilization step. To prevent unspecific binding, BSA/PBS (blocking buffer, 2% BSA) was used. As negative control, HUVEC were additionally incubated with endoN (7 µg/mL in blocking buffer for 1 h at 37 °C). PolySia was detected with mAb 735 (20 µg/mL in blocking buffer for 1 h at 37 °C) in combination with AlexaFluor^®^488 α-mouse IgG (7 µg/mL in blocking buffer for 45 min; ThermoFisher Scientific, Darmstadt, Germany). Slides were sealed with Vectashield^®^ antifade mounting medium with DAPI (Vector Laboratories, Burlingame, USA). Images were recorded with a Motic Mikroskop BA410 microscope equipped with a Moticam Pro 252B camera (Motic GmbH, Wetzlar, Germany).

### mRNA analysis

The transcript levels of ST8SiaII and ST8SiaIV were analyzed using first semi-quantitative reverse-transcriptase PCR (RT-PCR) to roughly estimate the transcript levels of ST8SiaII and ST8SiaIV and then fluorescence-based real-time quantitative RT-PCR was used to determine their exact copy numbers. Total RNA was isolated from homogenized HUVEC samples using the RNeasy Plus MiniKit (Qiagen, Hilden, Germany). Subsequently, cDNA was synthesized using the DNase1 Kit (Thermo Fisher Scientific, Darmstadt, Germany) and the iScript Kit (BioRad, Düsseldorf, Germany). Finally, ST8SiaII and ST8SiaIV transcripts were amplified using specific oligonucleotide primers (Table 1) and the OneTaq DNA Polymerase according to manufacturer’s instruction (New England BioLabs, Frankfurt a. Main, Germany). GAPDH (glyceraldehyde 3-phosphate dehydrogenase) was included as reference gene and *aqua dest*. was used as negative control. The cDNA was denatured for 30 s at 94 °C and then subjected to 40 cycles of each 30 s at 94 °C, 30 s at 52 °C and 1 min at 68 °C. Afterwards, samples were incubated for 5 min at 68 °C and loaded on a 1.5% gel containing ethidium bromide for electrophoretic separation and subsequent visualization of the specific PCR bands.

**Table 1:** Primers used in this study

Gene	Accession No.	Sense primer (5′–3′)	Antisense primer (5′–3′)	Amplicon length (bp)
Semiquantitative qPCR				
*GAPDH*	NM_002046	AGTCAACGGATTTGGTCGTA	ACCATGTAGTTGAGGTCAATGAAG	111
*ST8SiaII*	XM_017022642	CCAGCTGTTGTTGACAGAAGTAA	TAAAATCTGCTTCCTGATCCTC	111
*ST8SiaIV*	XM_011543630	CTTCCAGCACAATGTAGAAGGTTG	GCTCTTGACCACTGACACATCTC	112
Fluorescence-based RT-qPCR				
*GAPDH*	NM_002046	AAGATGCGGCTGACTGTCG	GTGACCAGGCGCCCAATAC	113
*ACTB*	NM_001101	TCCTGTGGCATCCACGAAACTA	CGGCAATGCCAGGGTACA	121
*EEF1A1*	NM_001402	AAAATGGGAAAGGAAAAGACTCA	GGGGCATCAATGATAGTCACA	281
*ST8SiaII*	XM_017022642	GGAGGCAGAGGTACAATCAGATCA	TTCTGTCAACAACAGCTGGTGAT	106
*ST8SiaIV*	XM_011543630	TTGTCTTTGAGTCGGTCACTTGT	TCTAGGACCAAAGAGGAATTGATT	116

In parallel, the fluorescence-based RT-qPCR was carried out using the LightCycler 96 system (Roche, Mannheim, Germany) combined with the Sensi-FAST SYBR No-ROX Kit (Bioline, Luckenwalde, Germany) and a different set of specific primers as used for semiquantitative qPCR (cf. Table 1). The LightCycler 96 program included an initial denaturation step (95 °C, 5 min), followed by 40 cycles of 5 min at 95 °C, 15 s at 60 °C and 15 s at 72 °C. The fluorescence was measured 10 s at 72 °C. The quality of the PCR products was assessed based on gel electrophoresis and melting-curve analysis. LightCycler data were analyzed using the LightCycler 96 analysis software v. 1.1. The copy numbers of ST8SiaII and ST8SiaIV were calculated on the basis of gene-specific standard curves (10^7^ to 10^3^ copies per 5 µL; *R*^*2*^ > 0.999) and normalized with a factor based on the geometric mean of the three reference genes GAPDH, ACTB (β-actin) and EEF1A1 (eukaryotic translation elongation factor 1 alpha 1).

### Scratch assay

To examine the migration capacity of HUVEC, scratch assay was performed [30, 31], using 12 well plates with 200,000 cells/well. The plates were marked on the outer bottom side of each well allowing to indicate comparable areas for scratching and to take photos of the same zone of each well. After reaching a confluent monolayer, cells were set into a steady non-proliferative state by changing medium from ECGM (including 10% FCS) to ECBM with 0.5% FCS and incubated for 24 h. Subsequently, scratches were placed into the monolayers using a 200 µL pipette tip. After a washing step using PBS, ECBM (0.5% FCS) (in the absence or presence of 1 µg/mL endoN) was added. Photos were taken with an inversed cell culture microscope (Hund, Type Wilovert 30, Wetzlar, Germany). The scratch area was evaluated and the relative distances of migrated cells were calculated.

### Native agarose gel-electrophoresis

For separation of proteins in native agarose gels, recombinant VEGF (Life Technologies, Carlsbad, California), histones from calf thymus (Sigma-Aldrich, Steinheim, Germany) or catalase from bovine liver (Serva, Heidelberg, Germany) were each incubated with polySia (colominic acid from *E. coli* equal to polySia in mammals; GERBU, Heidelberg, Germany) in 50 mM Tris buffer for 1 h at 30 °C with agitation. The samples were loaded onto a 2% agarose gel (peqGOLD Universal Agarose, peqLab, Erlangen, Germany) and separated using 19.2 mM glycine in 25 mM Tris/HCl (pH 8.5) buffer. The electrophoresis was run at 80 V (constant voltage) for 3.5 h [32]. Thereafter, the gel was fixed in 45% methanol containing 7.5% acetic acid (v/v) overnight. Roti-blue (Roth, Karlsruhe, Germany) was used as Coomassie blue staining-dye according to manufacturer’s instructions.

### Statistical analysis

Data sets were analyzed by paired Student-t test using GraphPad Prism Version 7.03. Differences were considered statistically significant at p < 0.05. Significant differences are given: *p < 0.05; **p < 0.01; ***p < 0.001; ****p < 0.0001.

## Results

### PolySia is released by cultured HUVEC

**Fig. 1:**
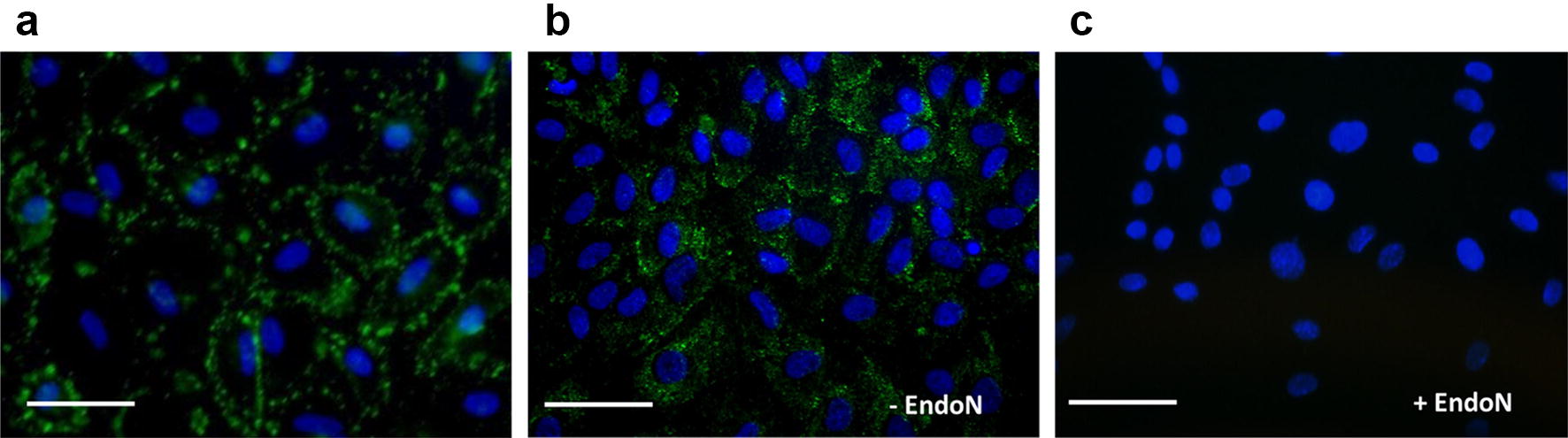
Distribution of polySia in HUVEC. **a** PolySia was visualized in unpermeabilized HUVEC using mAb 735 in combination with an Alexa Fluor labeled secondary antibody. **b** In addition, polySia staining was performed after fixation and permeabilization using ice cold ethanol. **c** To prove the specificity of the antibody recognition, in a parallel set HUVEC were treated with endoN to digest polySia chains. Scale bar 100 µm

PolySia is produced by endothelial cells, as already described by Naftolin and colleagues [24–26]. In order to explore possible polySia dependent mechanism in HUVEC, the polySia synthesis as well the location of polySia were characterized in more detail. In a first set of experiments polySia was visualized using HUVEC, which were fixed with 4% paraformaldehyde in PBS without a permeabilization step. Immunostaining with the mAb 735 against polySia revealed a distribution of sialic acid polymers in a punctuated pattern along cell–cell borders in quiescent, untreated cell monolayers (Fig. 1a). In contrast to unpermeabilized cells, polySia was additionally localized in granular patterns, mainly in association with intracellular vesicular structures, when HUVEC were permeabilized during fixation with ice cold ethanol (Fig. 1b). The specificity of the immune-staining was supported by preincubation of cell monolayers with endoN to remove polySia residues prior to incubation with the anti-polySia mAb 735 (Fig. 1c). Thus, polySia seems to be partially present in intracellular vesicles as well as on the surface of HUVEC.

**Fig. 2:**
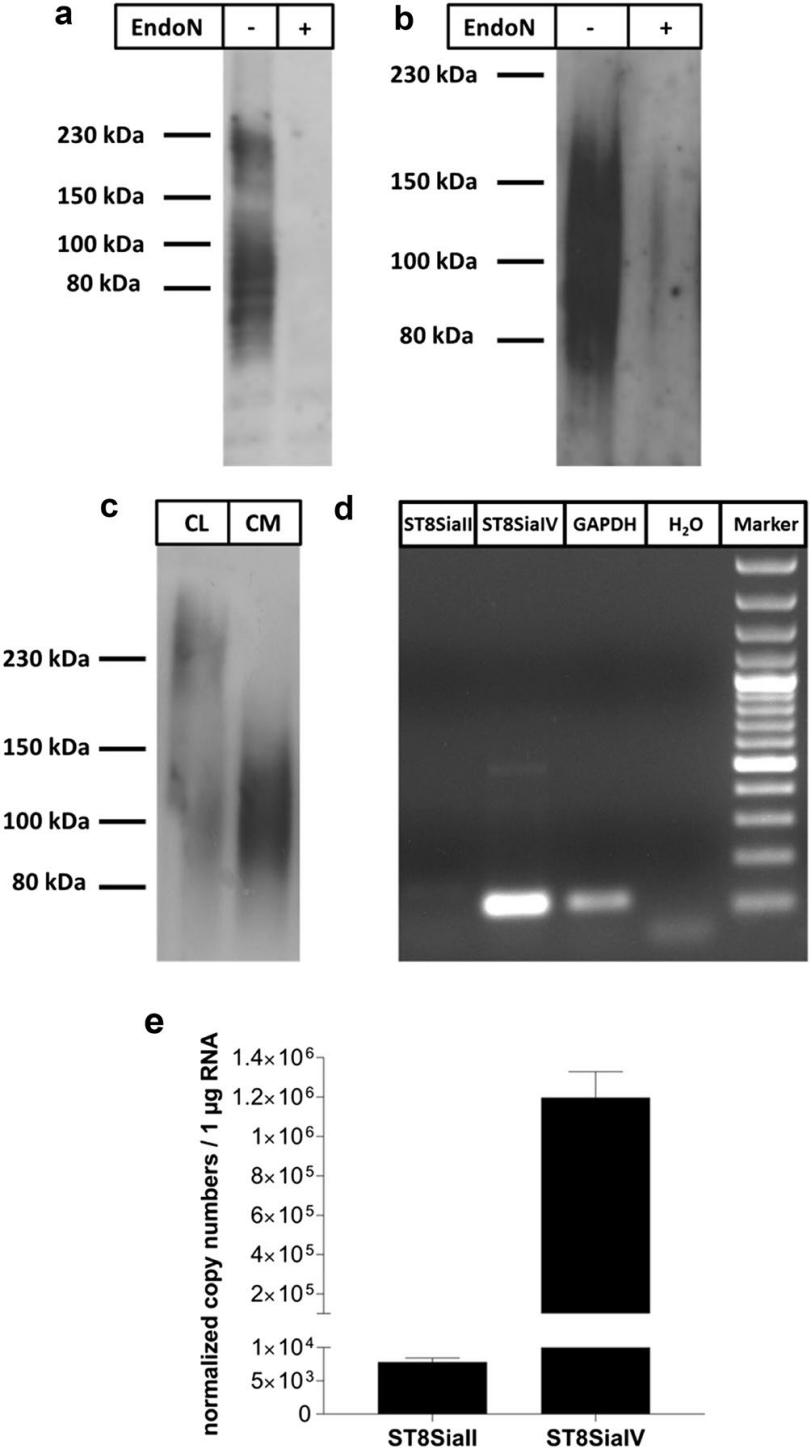
Detection of polySia in cell lysates and cell culture supernatants; mRNA levels of polysialyltransferases. **a** PolySia was visualized in cell lysates using mAb 735. For negative control, sample was treated with endoN. **b** PolySia was enriched from cell culture supernatants with the help of mAb 735-coupled beads, and subsequently analyzed by Western blotting using antibody against polySia. **c** After immunoprecipitation of polySia from cell lysate (CL) and the corresponding cell culture supernatant (CM), polySia was visualized by Western blotting. **d**, **e** Total RNA was isolated from HUVEC and reverse-transcribed to cDNA. **d** Semi-quantitative PCR was performed to visualize the abundance of ST8SiaII and ST8SiaIV transcripts. GAPDH was used as reference. One representative gel-electrophoresis picture is shown. **e** Fluorescence-based quantitative RT-PCR was used to determine the number of ST8SiaII and ST8SiaIV transcripts in HUVEC (normalized against GAPDH, ACTB and EEF1A1 and calculated to 1 µg of total RNA, which was used as template for cDNA synthesis). Error bars indicate the standard deviation (n = 6)

The presence of polySia in parallel samples could be confirmed by Western blotting using cell lysates of HUVEC (Fig. 2a). The detection of polySia bands by the mAb 735 indicated two broad signals in untreated samples, whereas no signals could be detected after endoN application, demonstrating the specificity of the signal.

In addition to the cell lysate, the cell culture medium was checked for the presence of sialic acid polymers by Western blotting. Following cultivation of HUVEC in FCS-free medium overnight, the carbohydrates were adsorbed onto beads coated with mAb 735. As shown in Fig. 2b, a strong polySia signal was detectable in cell culture supernatants. In contrast to cell lysates only one broad band was discernable in the range of 100 kDa.

To compare the signals of secreted polySia versus intracellular polySia levels in more detail, sialic acid polymers were isolated from cell lysates as well as from the corresponding supernatants using magnetic beads containing mAb 735, followed by Western blot analysis. The obtained results let suggest that the main pool of polySia was secreted and is present in the cell culture supernatant (Fig. 2c).

Two polysialyltransferases are able to generate sialic acid polymers in mammals, designated ST8SiaII and ST8SiaIV. In order to examine, whether both or only one polysialyltransferase is expressed in HUVEC, the presence of the respective mRNA was tested. Since mainly mRNA for ST8SiaIV was detectable in HUVEC (Fig. 2d), this polysialyltransferase appears to be responsible for the biosynthesis of polySia. We confirmed this initial observation using fluorescence-based RT-qPCR. Accordingly, the number of transcripts encoding ST8SiaIV exceeds that of ST8SiaII by a factor of 153 (Fig. 2e).

Taken together these results indicate that polySia is synthesized by ST8SiaIV and can be released by HUVEC.

### Degradation of polySia supports the migration of HUVEC

**Fig. 3:**
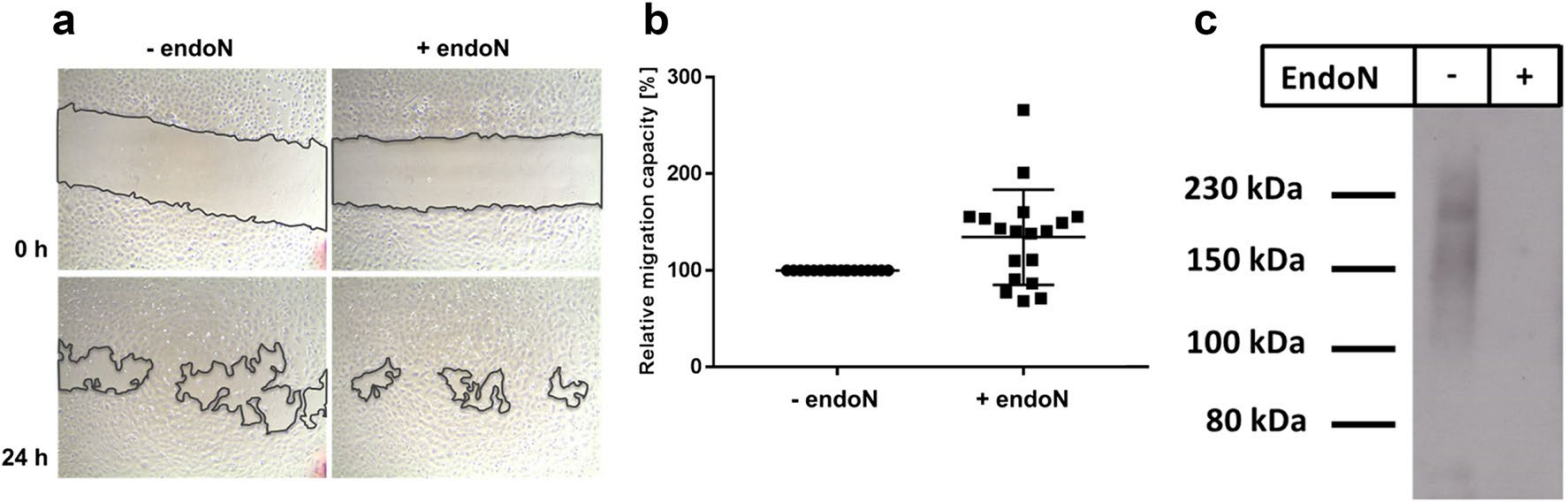
PolySia and cell migration. **a** HUVEC migration was examined by scratch assay in the absence (endoN treatment) or presence of polySia (no endoN). **b** Areas of untreated cell settings (control) were determined and set to 100%. Values are means of 18 scratches. The statistical evaluation was performed by paired Student’s t test (two tailed). Significance level is indicated by **p < 0.005. **c** The presence of polySia in the applied cell culture medium was assessed by immunostaining with anti-polySia antibody by Western blotting. Samples were pretreated with buffer (−) or endoN (+) before analysis

To test, whether polySia modulates the cellular migration of HUVEC, in vitro scratch assays were applied to confluent cell monolayers [30, 33]. Following scratching, the rate of closure (“wound healing”) of the resulting gap size was measured [34, 35]. In a parallel set of experiments, endoN was applied to remove extracellular polySia polymers, such that this condition can be considered as a polySia-negative control. As shown in Fig. 3a, b, an enhanced migration of HUVEC was observed, when polySia was digested with endoN. It needs to be noticed that the experiments were performed with ECBM containing 0.5% FCS. Since FCS also contains polySia (Fig. 3c), the observed differences after breakdown of polySia represent a combined degradation-effect of both polySia from HUVEC and FCS.

**Fig. 4:**
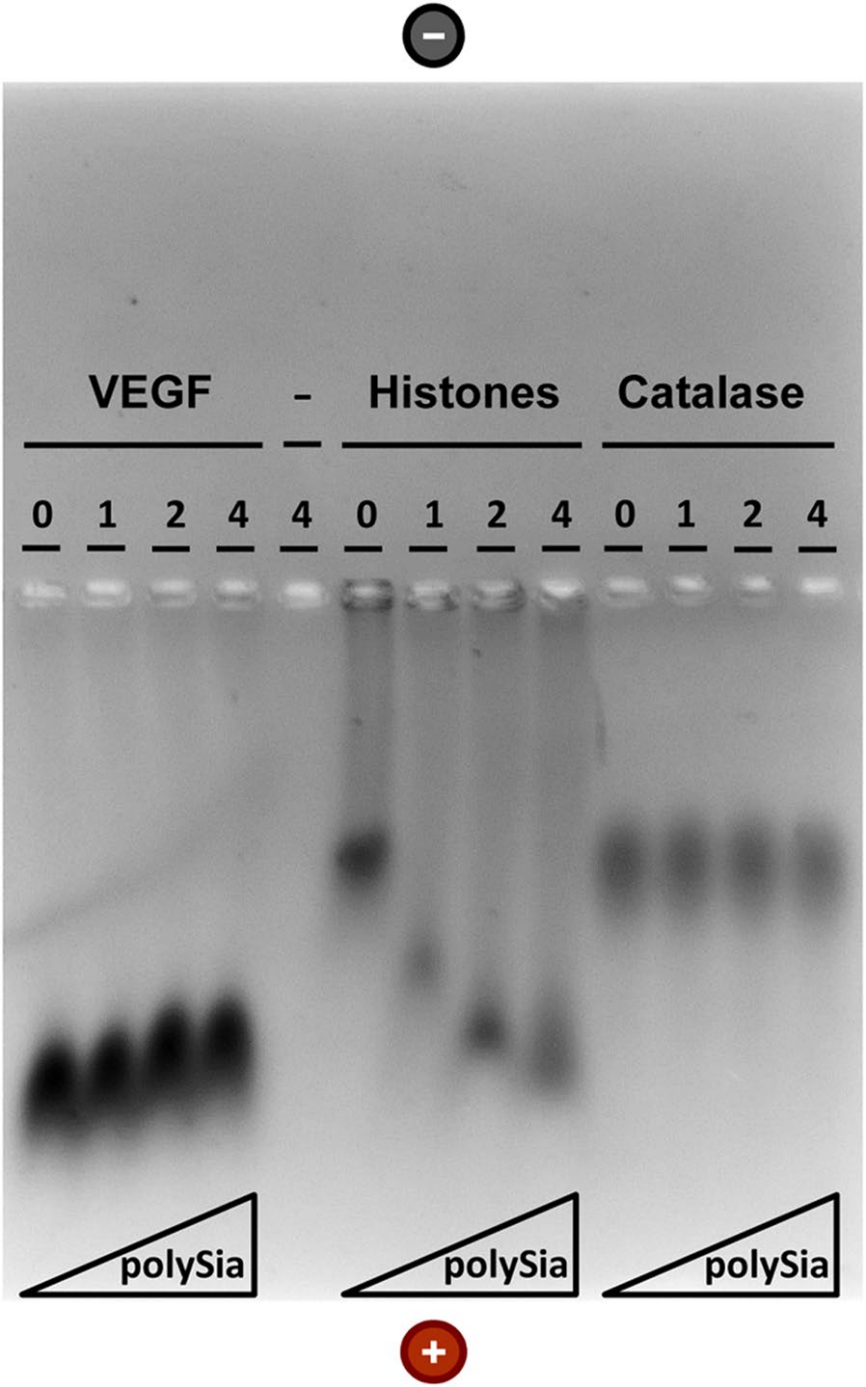
Interaction of polySia with VEGF. In binding assays, the indicated proteins (2 µg each) were incubated with increasing doses of polySia (1–4 µg/mL as shown), and the reaction mixtures were applied to native gel electrophoresis. The control lane (−) contained only polySia but no protein. Proteins were visualized by staining with Coomassie Blue

The migration of endothelial cells is regulated by cytokines [36–38]. Besides VEGF, bFGF is one of the best known modulators of HUVEC. Interestingly, bFGF was already shown to bind to polySia [22] and both cytokines, VEGF and bFGF, are persistently produced and secreted by HUVEC [39, 40]. We wanted to test, if also VEGF can interact with polySia. Utilizing native gel electrophoresis, the binding of VEGF to polySia was documented, resulting in a mobility shift of the complex as compared to the uncomplexed protein (Fig. 4). As a positive control for a polySia-complexing protein, a histone fraction was used that underwent mobility shift as well [41–43], whereas catalase as a negative control remained unchanged in its mobility during electrophoresis, despite the presence of polySia.

Based on these results, we propose that polySia influences the binding of cytokines like VEGF and bFGF to its receptor manipulating the migration capacity of HUVEC.

## Discussion

The carbohydrate moiety polySia is a key player during the pre- and postnatal development in mammals [7, 8, 10]. Besides its direct impact on cell adhesion as part of the glycocalyx, polySia is considered to influence e.g. cell differentiation and proliferation processes [7, 21, 44]. One example is the interaction of polySia with growth factors. Sato and co-workers observed that the functional activities of BDNF are enhanced particularly on polysialylated versus non-modified neuronal cells [23]. They proposed that polySia might concentrate BDNF on the cell surface thereby enhancing the recognition efficiency of BDNF by its receptor. Conversely, bFGF is captured by polySia, when this polymer is present on surface of neuronal cells, resulting in an inhibition of bFGF signaling [22]. This is in contrast to cell surface heparan sulfates that serve as co-receptors to concentrate and guide bFGF towards its cognate receptors [45]. Interestingly, polySia is also able to protect growth factors like bFGF and proBDNF against proteolytic cleavage [46]. In the case of bFGF only extended polySia chains showed this effect. Thus, in a chain length dependent manner the physiological activity of growth factors can be modulated by polySia.

Although endothelial cells express polysialylated glycoconjugates [24, 25], neither the expression analysis of polysialyltransferases nor a functional analysis of polySia has been performed. In line with previous studies of Naftolin and colleagues, an integration of polysialylated glycoconjugates was found within the glycocalyx as well as in a vesicular/granular pattern inside the cells [24, 25]. In addition, polySia was detectable as secreted form in the cell supernatants (Figs. 1 and 2). The results led us to propose that polySia-conjugates that are generated by ST8SiaIV in HUVEC need to become secreted via an as yet unknown mechanism.

**Fig. 5:**
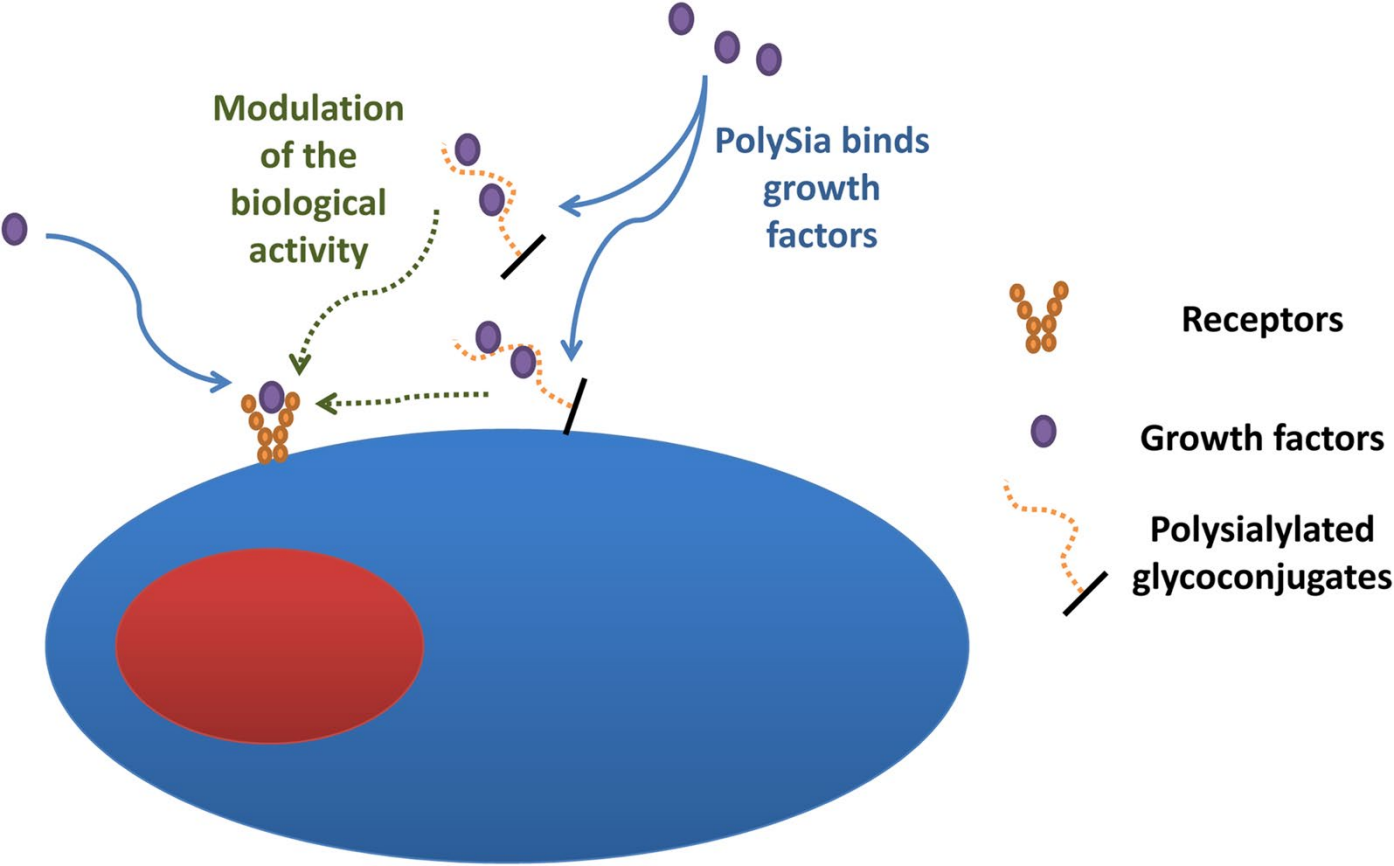
Working-model of polySia-dependent effect on HUVEC. Based on a model of Sato and colleagues [22], we hypothesize that polySia binds to growth factors also in the case of HUVEC. The trapping of growth factors by polySia might modulate their biological activities

In an approach to investigate a biochemical function of polySia, the degradation of polySia by endoN resulted in an increased migration capacity of HUVEC in vitro (Fig. 3b). Since VEGF and bFGF are produced and secreted by HUVEC [39, 40] and can be bound to polySia, we suggest that polySia may trap these growth factors modulating e.g. the binding between selected cytokines and their receptors (Fig. 5). Besides VEGF and bFGF also BDNF represents a further candidate, which might be involved, since also BDNF is known to influence angiogenesis [47]. To make it more complicated, polySia on the cell surface may influence the system in a different way than polySia in the supernatant. The outlined experiments demonstrate that polySia could be another player in the complex physiology of endothelial cells. However, further studies are necessary to get more insight into the polySia dependent mechanisms.

## Conclusion

The obtained results demonstrated that polySia in HUVEC is produced by polysialyltransferase ST8SiaIV and that a degradation of polySia increased the migration capacity of HUVEC. Based on our experiments showing an interaction between polySia and VEGF in addition to the already known interaction between polySia and bFGF as well as BDNF, we suggest that a complexation of these growth factors by polySia may modulate their physiological activity. Thus, besides neuronal cells [22], also in endothelial cells polySia might be a fine regulator of cellular processes.

## Abbreviations

bFGF: basic fibroblast growth factor
BDNF: brain-derived neurotrophic factor
endoN: endoneuraminidase
ECBM: endothelial cell basic medium
ECGM: endothelial cell growth medium
HUVEC: human umbilical vein endothelial cells
mAb: monoclonal antibody
NCAM: neural cell adhesion molecule
polySia: polysialic acid
RT-PCR: reverse-transcriptase PCR
VEGF: vascular endothelial growth factor
